# Glucose Metabolism and Oxidative Stress in Hepatocellular Carcinoma: Role and Possible Implications in Novel Therapeutic Strategies

**DOI:** 10.3390/cancers12061668

**Published:** 2020-06-23

**Authors:** Monica Mossenta, Davide Busato, Michele Dal Bo, Giuseppe Toffoli

**Affiliations:** 1 Experimental and Clinical Pharmacology Unit, Centro di Riferimento Oncologico di Aviano (CRO), IRCCS, 33081 Aviano (PN), Italy; monica.mossenta@cro.it (M.M.); davide.busato@cro.it (D.B.); gtoffoli@cro.it (G.T.); 2 Department of Life Sciences, University of Trieste, 34127 Trieste, Italy

**Keywords:** HCC, glucose metabolism, oxidative stress, tumor microenvironment, anticancer-immunoresponse

## Abstract

Hepatocellular carcinoma (HCC) metabolism is redirected to glycolysis to enhance the production of metabolic compounds employed by cancer cells to produce proteins, lipids, and nucleotides in order to maintain a high proliferative rate. This mechanism drives towards uncontrolled growth and causes a further increase in reactive oxygen species (ROS), which could lead to cell death. HCC overcomes the problem generated by ROS increase by increasing the antioxidant machinery, in which key mechanisms involve glutathione, nuclear factor erythroid 2-related factor 2 (Nrf2), and hypoxia-inducible transcription factor (HIF-1α). These mechanisms could represent optimal targets for innovative therapies. The tumor microenvironment (TME) exerts a key role in HCC pathogenesis and progression. Various metabolic machineries modulate the activity of immune cells in the TME. The deregulated metabolic activity of tumor cells could impair antitumor response. Lactic acid–lactate, derived from the anaerobic glycolytic rate of tumor cells, as well as adenosine, derived from the catabolism of ATP, have an immunosuppressive activity. Metabolic reprogramming of the TME via targeted therapies could enhance the treatment efficacy of anti-cancer immunotherapy. This review describes the metabolic pathways mainly involved in the HCC pathogenesis and progression. The potential targets for HCC treatment involved in these pathways are also discussed.

## 1. Introduction

Hepatocellular carcinoma (HCC) is the sixth most common cancer and the fourth leading cause of cancer-related death [1,2,3]. HCC develops in a multistep process, in the majority of cases arising on a background of liver damage [1,3,4,5].

Cancers have an altered metabolism with an increase in glucose uptake and lactate production in the presence of oxygen. This increased energy supplied by aerobic glycolysis is called “the Warburg effect” [6,7]. Oxidative stress is an important player in liver carcinogenesis [8,9,10,11]. It is caused by an imbalance between the production of oxidative species and antioxidant molecules in cells. The most abundant reactive species are the reactive oxygen species (ROS) [11]. The presence of elevated ROS levels causes damage to DNA with the subsequent production of 8-hydroxy-2-deoxyguanine and the increase of DNA mutations. Elevated ROS levels also cause damage to RNA; to lipids, with an increase in cell membrane permeability and cell death; and to proteins, with alterations in their structure and a subsequent change or loss of function [11]. ROS promote cellular proliferation, evasion of apoptosis, angiogenesis, tissue invasion, and metastasis [11].

Alterations in metabolic pathways could generate advantages for tumor proliferation and growth by ensuring energy and substrates. These alterations could create advantageous reducing conditions in the tumor microenvironment (TME) that are optimal for cancer survival [7,12].

In the current review, we discuss the role of glucose metabolism and oxidative stress in HCC pathogenesis and progression and the potential therapeutic targets for new HCC treatments.

## 2. HCC and Glucose Metabolism

### 2.1. Glucose Metabolism

**Figure 1:**
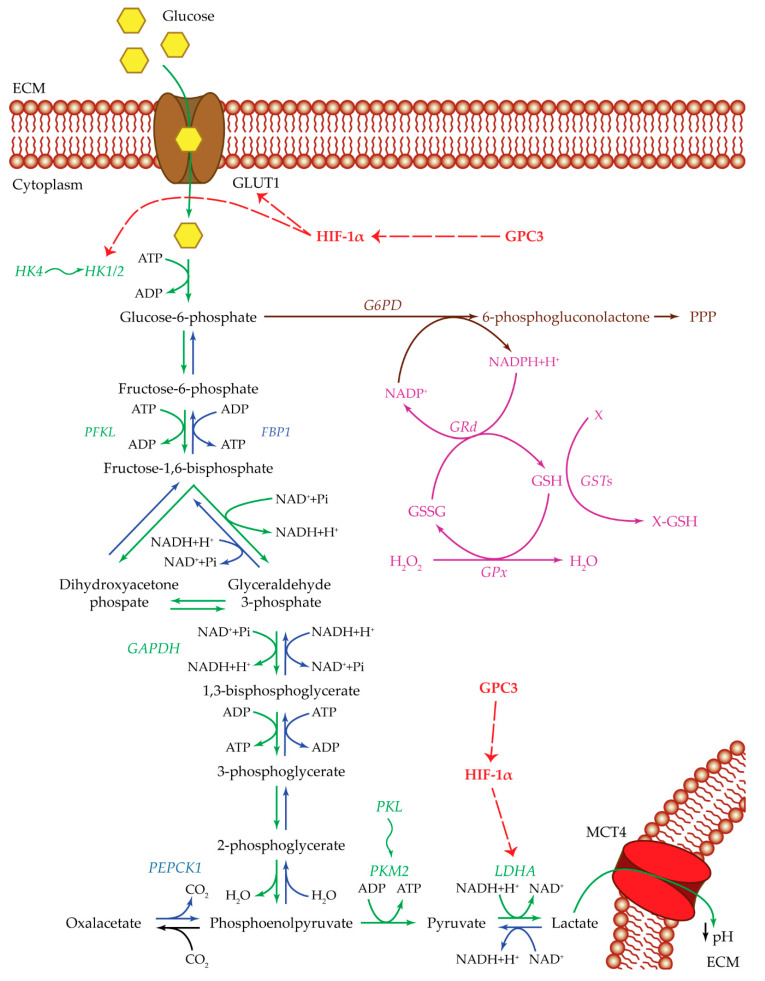
Glucose metabolism. Summarized are the most important players of the pathways used by cancer cells: glycolysis pathway (green), gluconeogenesis pathway (blue), pentose phosphate pathway (brown), and glutathione cycle (purple). Upregulating actions of GPC3 and HIF-1α are visualized with a red dotted arrow. Isoenzyme switches are illustrated as wavy arrows. HCC metabolism is shifted towards anaerobic glycolysis with an increase in glucose uptake by the activity of the GLUT1 transporter. Once inside the cell, glucose is transformed into G6P by HK1/2. Both GLUT1 and HK1/2 are positively regulated by HIF-1α, which in turn is upregulated by GPC3. In one case, G6P could be redirected towards the PPP, to produce metabolic intermediates useful for cell survival, and NADPH essential for glutathione reduction and ROS control. In the other case, G6P could continue through the anaerobic glycolytic pathway until the transformation of pyruvate into lactate by LDHA. The upregulation of LDHA enzyme is essential for the glycolytic pathway to remain active. This step could be regulated by HIF-1α and GPC3. In this way, cancer cells produce both energy and metabolic intermediates for all the macromolecular biosynthesis necessary for cell survival and proliferation. Lactate is then released out of the cell through the MCT4 transporter, ensuring an acidic pH in the extracellular compartment, which in turn maintains a state of inflammation and can modulate the immune system state of the tumor microenvironment. Abbreviations: ECM = extracellular matrix; FBP1 = fructose-1,6-bisphosphatase 1; G6PD = glucose-6-phosphate dehydrogenase; GAPDH = glyceraldehyde-3-phosphate dehydrogenase; GLUT1 = glucose transporter 1; GPC3 = glypican-3; GPx = glutathione peroxidase; GRd = glutathione reductase; GSH = glutathione reduced form; GSSG = glutathione oxidized form; GSTs = glutathione S-transferases; HIF-1α = hypoxia inducible factor 1α; HK1/2 or 4 = hexokinase 1/2 or 4; LDHA = lactate dehydrogenase A; MCT4 = monocarboxylate transporter 4; PEPCK1 = phosphoenolpyruvate carboxykinase 1; PFKL = phosphofructokinase L; PKL or M2 = pyruvate kinase L or M2; PPP = pentose phosphate pathway; X = oxidative stress by-product; X-GSH = oxidative stress byproduct bound to GSH.

HCC cell metabolism (Figure 1) is shifted to an increased glucose uptake through the glucose transporter 1 (GLUT1) channel [13,14,15]. *GLUT1* expression is significantly associated with HCC tumorigenicity, tumor invasiveness, and growth. GLUT1 protein expression is increased in HCC cancer tissues and is associated with an increase in ^18^F-FDG PET-CT (glucose analogue) uptake [13,14,15]. Once inside the HCC cells, glucose is converted into glucose-6-phosphate (G6P) by the activity of proteins belonging to the hexokinase (HK) protein family. It was demonstrated that HK2 is highly expressed in HCC, and it correlates with poor overall survival (OS) [16,17]. The HK family is formed by five major isoforms with tissue-specific profiles: HK1 is typical of brain and erythrocytes, HK2 is found in skeletal muscle and adipocytes, HK3 expression is low in most tissues, HK4 is typical of liver and pancreas, and the isoform hexokinase domain containing 1 (HKDC1) is typical of the gestational period [12,18,19,20]. HK1, HK2, and HK3 have a glucose affinity approximately 250-fold higher than that of HK4. During liver tumorigenesis, HK4 is silenced, and the high-affinity enzymes HK2, predominantly, and HK1, to a lesser extent, are activated [21].

At this stage, G6P could continue through glycolysis to produce ATP or can be redirected to the pentose phosphate pathway (PPP) to contribute to macromolecular biosynthesis [7]. These two different pathways are alternative and are activated simultaneously.

In the case of the glycolytic pathway, G6P is converted into fructose-6-phosphate, and subsequently into fructose 1,6-bisphosphate by the phosphofructokinase (PFK) enzyme. There are three PFK isoforms: PFKM, expressed in skeletal muscles; PFKL, typical of liver; and PFKP from platelets. *PFKL* gene expression was found to be increased in HCC tissues compared to adjacent non-tumor tissues [22,23,24]. The last steps are the conversion into glyceraldehyde-3-phosphate and to 1,3-bisphosphoglycerate thanks to glyceraldehyde-3-phosphate dehydrogenase (GAPDH), which is upregulated in HCC [25,26]. The last step in glycolysis is the production of pyruvate through one of the members of the pyruvate kinase (PK) family. The PK family groups four isoforms deriving from two paralogous genes: *PKL* and *PKM* [27]. The first one encodes the proteins PKL, expressed in normal liver and kidneys, and PKR, expressed in red blood cells. Meanwhile, *PKM* is subjected to splicing and generates two isoforms: PKM1, expressed in brain, bladder, adult muscle, and fibroblasts; and PKM2, expressed in tissues during embryogenesis and upregulated in multiple cancers [27,28]. PKM2 mRNA and protein were found to be overexpressed in HCC samples compared to healthy liver tissue and to be correlated with aggressive features and poor prognosis [12,27].

Pyruvate subsequently reacts with NADH and H^+^ in the presence of lactate dehydrogenase (LDH) to produce NAD^+^ and lactate [12]. Five LDH isoenzymes are present in human tissues. Each isoenzyme is a tetrameric enzyme composed of two subunits: M or A and H or B. The M subunit is typical of skeletal muscle, while the H is predominant of heart muscle. A disequilibrium towards the B chains (LDHB) causes a conversion of pyruvate into acetyl-CoA, while an increase in the A subunit (LDHA) drives the conversion of pyruvate into lactate. LDHA is the predominant isoenzyme in cancer cells [29]. The produced lactate is then released in the extracellular compartment through the monocarboxylate transporters (MCT) [12]. The production of lactate is essential to the glycolytic pathway remaining activated [7]. LDH is a key factor for HCC tumor proliferation and invasion. Analysis of HCC patients’ serum samples suggested the use of serum LDH as a prognostic factor [30,31,32,33,34,35]. High levels of MCT4 and CD147, a transmembrane glycoprotein able to induce matrix metalloprotease (MMP) production and to interact with MCT4 for lactate secretion, were detected in HCC tissues [36,37,38,39].

In the pathway alternative to glycolysis, G6P is redirected towards the PPP for macromolecular biosynthesis, and it is converted to 6-phosphogluconolactone by the glucose-6-phosphate dehydrogenase (G6PD), which was found to be upregulated in HCC [7,12,40,41,42]. The reaction catalyzed by G6PD produces NADPH, which is then used to reduce glutathione from GSSG form to GSH [12,43].

Conversely to glycolysis, the gluconeogenesis pathway is downregulated in HCC [12,44]. Fructose-1,6-bisphosphatase (FBP) is a rate-limiting enzyme able to convert fructose 1,6-bisphosphate into fructose 6-phosphate, the precursor of G6P. Two FBP isoforms are present in mammals: FBP1, typical of liver and kidneys; and FBP2, initially found in muscle tissues, and recently detected in all cells [12,45]. A significantly decreased expression of *FBP1* was found due to copy number loss or promoter methylation in HCC cases, suggesting that the related FBP1 protein is crucial for HCC tumorigenesis [44]. 

The nuclear receptor Nur77, encoded by the *NR4A1* gene, regulates gluconeogenesis by upregulating the transcription of *FBP1* and *FBP2* in the liver [46]. It also acts as a tumor suppressor capable of interacting with phosphoenolpyruvate carboxykinase (PEPCK1), impeding its degradation and, consequently, promoting the gluconeogenesis pathway and the conversion of oxalacetate to phosphoenolpyruvate [46]. There are two PEPCK isoforms: PEPCK1 or PEPC-C, which is the cytoplasmic form, highly expressed in the liver, kidneys, and adipose tissue; and PEPCK-M, which is the mitochondrial isoform [46]. In HCC samples, Nur77 expression was found to be decreased from Stage I to III [46]. A similar trend was found for PEPCK1 expression levels [46,47].

Another player that regulates glycolysis and gluconeogenesis is the AMP-activated protein kinase (AMPK). AMPK is an intracellular energy sensor that exerts a key role in the management of metabolism and cell growth [48]. In physiological conditions, it regulates the amount of ATP, restoring its level when necessary [48]. AMPK activation prompts the switch from glycolysis toward oxidative phosphorylation, and hence the inhibition of “Warburg effect” [49]. AMPK activation represses the production of DNA, RNA, proteins, and lipids fundamental for cell proliferation and expansion [49].

AMPK can have an impact in HCC tumorigenesis and progression [50]. AMPK activity is downregulated in malignant regions compared to their non-malignant counterparts. This decreased activity of AMPK is correlated with worst prognosis [51,52]. AMPK activation stimulates cell cycle arrest, regulates the survival of cancer cells, and diminishes the metastatic potential of tumor cells [50]. The silencing of AMPK inhibits apoptosis and cell cycle arrest caused by the starvation of glucose [53]. Several AMPK activators were studied for HCC treatment. The most studied is metformin, an AMPK activator anti-diabetic drug [49]. The activation role of metformin is exerted by impacting the AMP/ATP ratio and the mitochondrial respiration complex I, and stimulating the activation of liver kinase B1 (LKB1) [54,55]. Several studies demonstrated a reduced HCC incidence in Type 2 diabetic patients administered metformin [49]. In vitro and in vivo studies demonstrated that metformin can decrease the proliferation of HCC cells [51]. Three clinical trials are currently evaluating metformin in HCC (NCT03184493, NCT04033107, NCT04114136).

### 2.2. Hypoxia and Glucose Metabolism

Hypoxia-inducible factors (HIFs) represent important sensors for intratumoral oxygen concentration. The hypoxia system is based on two groups of HIFs, the α-subunits or HIFα which includes HIF-1α, HIF-2α, and HIF-3α, and the β-subunits or HIFβ which includes HIF-1β, and the aryl hydrocarbon receptor nuclear translocator 2 and 3 (ARNT2 and ARNT3) [56]. In normal oxygen conditions, HIF-1α undergoes prolyl hydroxylation, then it is recognized by the E3 ligase called von Hippel–Lindau tumor suppressor protein (VHL), and finally it is constitutively degraded by the proteasome [56]. In hypoxic conditions, the lack of a sufficient oxygen supply causes an impairment in HIF-1α hydroxylation and degradation. This leads to HIF-1α stabilization and translocation into the nucleus, where it heterodimerizes with HIF-1β and binds to the core hypoxia-response elements [56]. HIF-1αβ plays crucial roles in angiogenesis, proliferation, invasiveness, and cancer metabolism [57]. One of the major targets of HIF-1αβ is the vascular endothelial growth factor (VEGF). HIF-1αβ also acts on angiogenesis by producing nitric oxide synthase, endothelin-1, angiopoietin 2, and other molecules [57]. HIF-1αβ regulates proliferation by inducing Myc proto-oncogene protein (c-Myc), inhibitor of differentiation 2, and insulin-like growth factor-2 [57].

Hypoxia is a peculiar condition of HCC caused by the fast growth of tumor cells, with the consequent consumption of oxygen associated with an inadequate vascularization [58]. Studies on HCC patients’ tissue samples revealed a correlation between HIF-1α and/or HIF-2α and prognosis [59,60]. 

Data obtained in in vivo HCC models showed a significant correlation between microvessel density and both HIF-1α and VEGF expressions [61,62].

An in vitro study on HCC cell invasiveness showed that basil polysaccharide can inhibit CoCl_2_-based HIF-1α induction in HCC cell lines, rescuing the epithelial–mesenchymal transition (EMT) phenotype [63].

At low oxygen levels, the electron transport chain has an unbalanced flow of electrons with the consequent production of ROS which hampers cell survival [64]. To overcome ROS production, HIFs are activated and allow the transcription of genes that enhance glycolysis in anaerobic conditions, such as *GLUT1*, *HK2*, and *LDH* [13,58,64,65,66,67].

An increase in HIF-1α protein expression and in *GLUT1* mRNA expression was shown after hypoxia induction [13]. Another player of the glycolytic pathway that is regulated by hypoxia is HK2. HIF-1α can induce HK2 expression in human HCC cells. Treatment with HK2 or HIF-1α inhibitors suppressed cellular proliferation [65]. HK2 and HIF-1α immunostaining co-localized near necrotic regions in HCC tissue samples of patients pre-treated with transcatheter arterial embolization (TAE) [66].

HIF-1α can also be regulated by NANOG. The transcription factor and stem cell marker NANOG is involved in the sustenance of self-renewal, pluripotency, and the undifferentiated phenotype of stem cells [68]. NANOG can be activated by hypoxia, via HIF-1α, or by Toll-like receptor 4 (TLR4) [69,70]. In HCC, the induction of NANOG, mediated by TLR4, inhibits mitochondrial oxidative phosphorylation and stimulates fatty acid oxidation; these mechanisms prompt an inhibition of oxygen consumption rate and ROS production [70]. This scenario induces drug resistance and the sustenance of tumor-initiating stem-like cell self-renewal [70]. The re-establishment of oxidative phosphorylation and suppression of fatty acid oxidation could abrogate this situation of drug resistance [70].

HIF-1α binds the promoter region of lncRNA RAET1K, increasing its expression under hypoxic conditions [67]. RAET1K, in turn, is responsible for a decrease of miR-100-5p levels. In this way, the activity of this miR-100-5p in the decrease of lactate concentration and glucose uptake is diminished. Silencing of lncRNA RAET1K can suppress HCC cell proliferation and invasion [67].

### 2.3. Glypican 3 (GPC3) and Glucose Metabolism

GPC3 is a heparansulfate proteoglycan proposed as a target protein for HCC treatment for its high and specific expression on HCC cell surfaces [71,72,73,74,75,76,77].

GPC3 is involved in the reprogramming of tumor cell metabolism by acting on the glucose pathway (Figure 1) [36,37,78,79,80]. HCC cells, in presence of high GPC3 protein expression, have a higher glucose uptake and lactate production [78]. GPC3 can enhance HIF-1α protein expression. Knockdown of GPC3 expression was associated with tumor growth suppression, which was significantly reversed forcing the expression of HIF-1α [78]. A significant positive correlation was found between GPC3 expression and GLUT1, LDHA, and HK2 expression [78].

GPC3 may interact with MCT4 and GLUT4 on the cell surface, facilitating the adaption of HCC cells to the hypoxic environment. In addition, CD147, which is frequently co-localized with MCT4, might increase tumor invasiveness, inducing expression of MMPs [36,37]. 

An inverse correlation between GPC3 expression and ^18^F-FDG-6-phosphate uptake was found in HCC patients’ samples [79]. This inverse association might be caused by an inverse trend of GPC3 and GLUT1 expression [79]. A decrease in glucose uptake and GLUT1 protein expression was also found after GPC3C-terminal domain overexpression [80].

## 3. HCC and Oxidative Stress

### 3.1. Oxidative Stress

The oxidative metabolism of aerobic respiration by the mitochondria is the main source of oxidative stress [11]. The reactive species are ROS (the most abundant), RNS (reactive nitrogen species), RSS (reactive sulfur species), and RCS (reactive chloride species). When an unbalanced equilibrium towards ROS formation is present, a series of damages affects DNA, RNA, lipids, and proteins [11].

**Figure 2:**
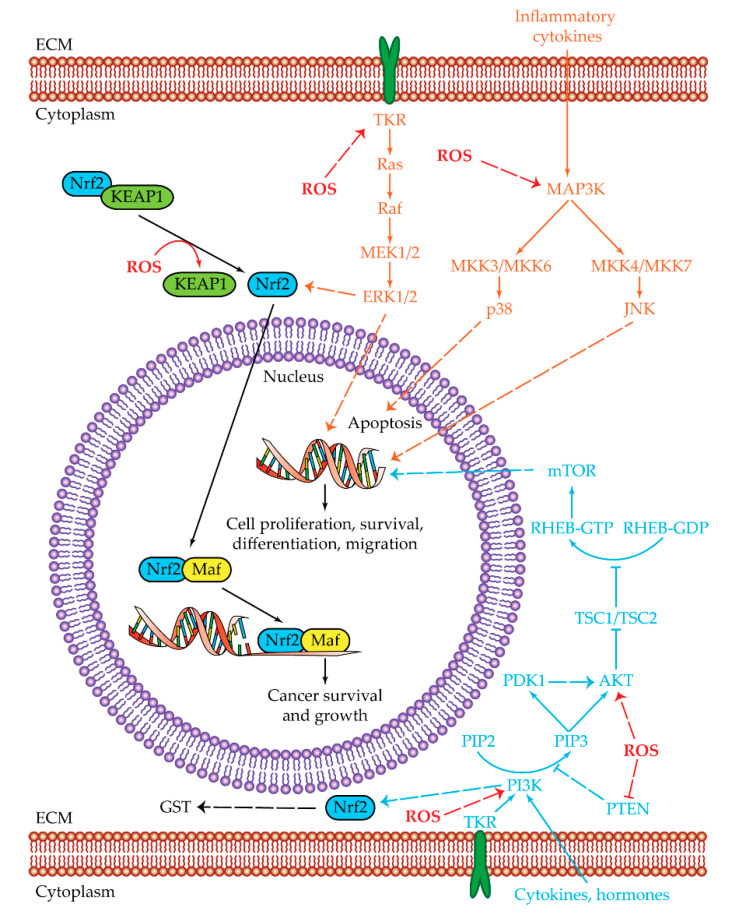
Oxidative stress pathways. Summarized are the two main pathways of cell proliferation and survival subjected to reactive oxygen species (ROS) regulation: MAPK (orange) and PI3K/AKT/mTOR (light blue). The shared Nrf2 pathway is highlighted in black, while ROS regulations/interactions are visualized in red. Positive regulations are pictured as dotted arrows, and negative regulations are pictured as dotted truncated arrows. High oxidative stress is one of the key aspects that in normal conditions leads to cell death. HCC cells can manage ROS overproduction by activating the MAPK and PI3K/AKT/mTOR pathways. The last effectors of MAPK pathways are ERK1/2, JNK, and p38. ERK1/2 and JNK are upregulated and lead to the transcription of genes involved in cell proliferation, survival, differentiation, and migration, while p38 is related to apoptosis and in HCC cells is downregulated. The PI3K/AKT/mTOR pathway is upregulated in HCC cells leading to cell proliferation, survival, differentiation, and migration. ROS can regulate PI3K and AKT activities by increasing their phosphorylation or by decreasing PTEN levels. Both MAPK and PI3K/AKT/mTOR pathways have a common effector which is Nrf2. Nrf2 is a transcription regulator which is maintained at low levels by the KEAP1 protein. In the presence of high ROS levels, KEAP1 dissociates from Nrf2, which by this dissociation becomes capable of reaching the nucleus. Once inside the nucleus, Nrf2 heterodimerizes with Maf. The Nrf2/Maf heterodimer binds the antioxidant-responsive elements for the transcription of genes involved in cancer cell survival and growth. Nrf2 is also able to activate genes related to oxidant homeostasis such as glutathione S-transferase (GST). Abbreviations: AKT = protein kinase-B; ECM = extracellular matrix; ERK1/2 = extracellular regulated kinases 1 and 2; JNK = c-Jun N-terminal kinases; KEAP1 = Kelch-like ECH-associated protein 1; GST = glutathione S-transferase; Maf = musculoaponeurotic fibrosarcoma protein; MAP3K = MAPK kinase kinase ; MEK1/2 = MAP/ERK kinases 1 and 2; MKK3/6 and MKK 4/7 = mitogen-activated protein kinase kinase 3/6 and 4/7; mTOR = mammalian target of rapamycin; Nrf2 = nuclear factor erythroid 2-related factor 2; p38 = p38 mitogen-activated protein kinases; PDK1 = serine/threonine kinase phosphoinositide-dependent kinase 1; PI3K = phosphatidylinositol 3-kinase; PIP2 = phosphatidylinositol 4,6-bisphosphate; PIP3 = phosphatidylinositol 3,4,5-triphosphate; PTEN = phosphatase and tensin homologue; Raf = rapidly accelerated fibrosarcoma protein; Ras = rat sarcoma protein; RHEB = Ras homolog enriched in brain; ROS = reactive oxygen species; TKR = tyrosine-kinase receptor; TSC1/TSC2 = tuberous sclerosis proteins 1 and 2 complex. 3.2. Nrf2 and Oxidative Stress.

ROS imbalance can promote tumor development and progression. There are two major pathways that can be regulated by ROS: the mitogen-activated protein kinase (MAPK) pathway and the phosphatidylinositol 3-kinase/protein kinase-B/mammalian target of rapamycin (PI3K/AKT/mTOR) pathway (Figure 2) [81,82].

The MAPK pathway regulates cell growth, differentiation, and apoptosis through a phosphorylation cascade that can start from tyrosine-kinase receptors (TKRs), and activates downstream protein effectors Ras, Raf, MAP/ERK kinases (MEK1/2), and extracellular regulated kinases (ERK1/2) [81]. Another mode of MAPK pathway activation is represented by cytokine activation with the subsequent phosphorylation of MAPK kinases (MAP3Ks), and phosphorylation of p38 mitogen-activated protein kinases (p38) or of c-Jun N-terminal kinases (JNK) [81,83].

ERK1/2 are required for G1/S phase transition after mitogen action. ERK1/2 are involved in the induction of proliferation, in the suppression of apoptosis, and in the promotion of cell survival [83]. MEK/ERK activation is fundamental for HCC cell proliferation [84,85]. Abnormal activation of the Ras/Raf/MEK/ERK signaling pathway was detected in HCC patients’ samples [86,87].

JNK has a role in cell cycle progression, cell proliferation, and migration [83]. Immunohistochemical staining of HCC samples revealed a positive signal for phosphorylated JNK1 (pJNK1) and JNK2 (pJNK2) in the majority of them [88]. JNK1 can promote HCC cell survival by increasing c-Myc levels [88]. High expression levels of pJNK/c-Jun (a JNK downstream effector) protein were associated with a lack of response to sorafenib treatment [89].

Gankyrin is an oncoprotein highly expressed in HCC, with an active role in tumor initiation and progression [90]. It enhances the production of lactate and glutamate and the employment of glucose and glutamine in HCC [91]. These activities are exerted through an increased expression of factors implicated in glutaminolysis and glycolysis. Gankyrin pilots glutaminolysis and glycolysis via the upregulation of c-Myc caused by β-catenin signaling activation. This metabolic reprogramming is c-Myc-mediated. It could contribute to tumor initiation, progression, and chemoresistance [91]. The use of c-Myc inhibitors contributed, with sorafenib or regorafenib, to tumor suppression in HCC patient-derived xenograft models with high levels of Gankyrin [91].

p38α protein regulates the production of pro-inflammatory cytokines, controls differentiation and apoptosis, and negatively regulates proliferation [83]. Mice presenting deletions in p38α showed an increase in liver cancer development, which was reduced by JNK/c-Jun inhibition [92]. Downregulation of p38 and MKK6 activities were associated with HCC lesions with a higher size [93].

The PI3K/AKT/mTOR pathway can be activated by TKRs, cytokines, and hormones or by Ras [94,95]. Once PI3K is activated it phosphorylates the phosphatidylinositol 4,6-bisphosphate (PIP2) into phosphatidylinositol 3,4,5-triphosphate (PIP3), which in turn activates both serine/threonine kinase phosphoinositide-dependent kinase 1 (PDK1) and AKT. AKT inhibits the tuberous sclerosis proteins 1 and 2 complex (TSC1/TSC2). As a consequence, the Ras homolog enriched in brain (RHEB) can be phosphorylated and can activate mTOR, which is involved in the induction of cell survival, proliferation, and protein translation [82,94]. AKT inhibits apoptosis by interacting with Bcl-2 associated agonist of cell death (BAD) and bcl-2-like protein 4 (BAX) or by preventing mouse double minute 2 homolog (MDM2) phosphorylation, which in turn inhibits cellular tumor antigen p53 (p53) [82,94]. AKT is also able to increase glycolysis, acting on GLUT1 activation and phosphorylating HK2 [82]. ROS can act on cell proliferation by increasing PI3K or AKT phosphorylation or by decreasing phosphatase and tensin homologue (PTEN) levels [82]. In HCC tissues, the PI3K/AKT/mTOR pathway was found to be upregulated, with PTEN loss and AKT activation correlated with poor differentiation, high proliferation, and intrahepatic metastasis [96].

The PI3K/AKT pathway is affected by mutations in HCC. One mutated gene is *PIK3CA*, which encodes for the catalytic subunit of the PI3K protein. The *PIK3CA* mutation frequency is about 7%. *PIK3CA* mutations can be found in the helical domain (E545K) or in the kinase domain (H1047R) [97,98,99,100]. The mutation H1047R increased the AKT activity in HCC tissue compared to patients’ normal liver tissue [97,98,99,100]. SGK3 is a protein kinase downstream mediator of PI3K pathway [100]. Its silencing reduced HCC cell proliferation rate based on the overexpression of PIK3CA E545K, and not on PIK3CA H1047R overexpression. Using an in vivo E545K/c-Met HCC model, a delayed tumor formation in the presence of *SGK3* gene deletion was shown [100]. Activating mutations in *PIK3CA* may predict sensitivity to inhibitors of the PI3K/AKT mTOR pathway [99]. RSK2 is a serine/threonine kinase that regulates PI3K/Ras signaling and acts to provide negative feedback on the ERK1/2 pathway. A total of 9.6% of HCC samples are mutated in its gene *RPS6KA3*, and half of these mutations cause a premature stop codon or an altered splicing. To a lesser extent, missense mutations were found in close proximity to codons encoding for Ser227 or Thr557 phosphorylation sites required for RSK2 activation and thus inactivating its function [101,102].

Cancer cells, in order to reduce the overproduction of ROS and the related toxic effects while maintaining cell proliferation and survival conditions, can activate two major response mechanisms: the Kelch-like ECH-associated protein 1 (KEAP1)-nuclear factor erythroid 2-related factor 2 (Nrf2) (encoded by *NFE2L2* gene) pathway and GSH metabolism [82]. A minor mechanism able to reduce oxidative stress takes advantage of aldehyde dehydrogenase (ALDH) [103].

Nrf2 is part of the basic region leucine zipper transcription factor cap ‘n’ collar subfamily [104]. It binds the antioxidant-responsive elements (ARE) and activates genes involved in oxidant homeostasis and drug metabolism control, such as glutathione S-transferase (GST), glutathione peroxidase (GPx), and drug-metabolizing enzymes [11,104,105]. In cancer cells, Nrf2 is involved in the control of oxidative stress and in maintenance of cell survival and proliferation [104,105,106]. It can be activated by MAPK and PI3K pathways or directly by oxidative stress (Figure 2) [105,106]. In the latter case, some KEAP1 cysteines are oxidized and dissociated from Nrf2, which in turn can translocate into the nucleus. In this subcellular localization, Nrf2 dimerizes with musculoaponeurotic fibrosarcoma (Maf) protein. The Nrf2/Maf dimer binds ARE sequences in order to activate the transcription of enzymes of the phase II detoxification and of transporters [105,106]. In cancer, Nrf2 and its downstream genes are overexpressed, leading to cell survival and growth [106]. In HCC samples, Nrf2 is overexpressed and localized in the nucleus, suggesting its constitutive activation [106].

Nrf2 can be also activated by a positive feedback made with TRIM25, a member of the tripartite motif-containing family, which is upregulated during endoplasmic reticulum (ER) stress [107]. Specifically, TRIM25 targets KEAP1 and causes its ubiquitination and degradation. In this way, Nrf2 can reach the nucleus and activate genes, including *TRIM25* itself, to reduce ROS [107].

Somatic mutations that interfere with Nrf2–KEAP1 interaction were identified in HCC cases [108,109]. These mutations cause an increased transcriptional activity of Nrf2, stimulating tumorigenesis and cell proliferation [108]. A whole-exome sequencing conducted on 24 HCC cases revealed that 6.4% of them had mutated *NFE2L2* [102]. The DLG and ETGE motifs located within *NFE2L2* harbored these mutations that cause the inhibition of the binding of KEAP1 [102]. Another whole-exome sequencing analysis performed on 363 HCC cases showed *NFE2L2* mutations in 3% and *KEAP1* mutations in 5% of the cases, respectively [110]. A chemically induced HCC rat model demonstrated that *NFE2L2* mutations are very recurrent and they occur in the early stages of tumorigenesis, suggesting an active role in HCC activation and development [109]. *NFE2L2* knockout mice were able to withstand diethylnitrosamine-induced hepatocarcinogenesis [111]. This resistance to tumorigenesis was associated with the decreased expression of PPP-associated enzymes. These results showed that Nrf2 contributes to tumorigenesis, by upregulating the genes related to glucose uptake and reallocation within PPP [111].

### 3.2. Glutathione (GSH) and Oxidative Stress

GSH is a natural non-enzymatic and endogenous antioxidan [11,112]. It is composed of three peptides, L-γ-glutamyl-L-cyteinyl-glycine, and the most common forms are reduced GSH and oxidized GSSG. GSH is involved in cellular growth functions and it is employed in reactions carried out by other enzymes to detoxify several substrates in the phase II of detoxification [113,114,115]. GSH and some GSH-related enzymes were associated with tumor chemoresistance, given their ability to bind or interact with drugs, and their depletion enhanced the cytotoxic effects through oxidative stress [115,116]. In HCC tissues, GSH levels were found to be increased, making it a possible target for cancer therapeutics [114,117].

GSH is produced through two steps requiring ATP. One step is the reaction of glutamate and cysteine catalyzed by γ-glutamylcysteine synthetase (GCS) to produce γ-glutamylcysteine; the other step is the reaction of γ-glutamylcysteine with glycine catalyzed by GSH synthetase (GS) to produce GSH [114]. GCS is composed of the heavy catalytic subunit (GCS-HS) and the light subunit (GCS-LS) [114]. Once GSH is produced, it is used by the GSTs, which are phase II detoxification enzymes, to detoxify electrophilic xenobiotics and oxidative stress byproducts [117,118]. Other enzymes involved in the antioxidant machinery are GPx, which is able to convert H_2_O_2_ and GSH into H_2_O, O_2_ and GSSG, and glutathione reductase (GRd) which reduces GSSG to GSH again [115,117].

Huang et al. detected a 2-fold increase in the GSH protein level, and an increase in GCS-HS mRNA and protein level and in GS mRNA level in HCC tumor samples when compared to the normal liver counterparts [114]. This increment of GSH concentration was correlated with enhanced cell proliferation [114].

In HCC tissues, higher GSH and GPx activity were detected when compared to the adjacent normal liver counterpart. GSH and GSSG levels detected in pre-operative plasma were low, while after resection, their concentration increased, suggesting that GSH may be used to decrease the oxidative stress during carcinogenesis and that tumor tissue may uptake GSH from plasma [117]. 

### 3.3. Aldehyde Dehydrogenase and Oxidative Stress

Aldehyde dehydrogenases are a group of enzymes that catalyze the oxidation of endogenous and exogenous aldehydes, and are also necessary for the production of retinoic acid [103]. ALDH, by metabolizing a broad variety of aldehydes, decreases the levels of oxidative stress. In detail, reactive aldehydes (such as 4-hydroxy 2-nonenal), which can be produced after alcohol or anticancer drug catabolism or exposure to UV radiation or environmental pollutants, are converted into non-reactive species by members of ALDH family [103,119]. The high levels of ALDH expression in cancer cells, and in cancer stem cells, generate chemoresistance caused by the oxidation of the aldehyde group of the drug [103]. The administration of ALDH inhibitor sensitizes the cells to the anticancer treatment [103,120].

## 4. Immunological Reprogramming of the TME

In the HCC TME, immune system cells can be affected by metabolites released from cancer cells, in particular lactic acid–lactate [12,121,122,123]. Tumor-secreted lactate is employed by tumor-associated macrophages (TAM) to enhance the expression of VEGF and to prompt M2 phenotype [12,124,125,126,127,128].

Another type of immune cells that can be affected by lactate are the myeloid-derived suppressor cells (MDSCs) [121,129]. In a cancer context, the high expression of inducible nitric oxide synthase (iNOS), Arginase 1, and the generation of nitric oxide and ROS prompt the population of immature myeloid cells (IMC) to generate an immunosuppressive population of MDSCs [129]. The deficiency of LDHA results in lower MDSC presence [121]. NK cells can also be influenced by lactate [121].

Tumors can escape from dendritic cells (DCs) immunosurveillance by responding to several signals [122,130], including lactic acid, which interferes with differentiation and activation of monocyte-derived DC [122,131].

Cytotoxic T lymphocytes (CTL) can be affected by lactic acid, which is capable of inhibiting their cell growth and cytokine secretion [123,132].

Adenosine (ADO) is derived from ATP catabolism [133]. During cancer development, ATP is secreted at high levels by inflammatory, necrotic, or apoptotic cells [134]. During anti-cancer therapy, ATP can contribute to the elimination of cancer cells by inducing immunological cell death (ICD) [135]. In physiological conditions, ADO exerts mainly anti-inflammatory functions stimulating immune suppression and cytoprotection [133,136]. ADO is present at high levels in TME. ATP is secreted in response to hypoxia, which in turn induces the expression of HIF-1α, which drives the expression of CD39 and CD73 on tumor cells and on several immune components of the TME [133]. Adenosine diphosphate (ADP) and ATP are converted to adenosine monophosphate (AMP) by a reaction catalyzed through CD39, whereas CD73 catalyzes the conversion of AMP into ADO [133,137,138].

High ADO levels in the TME induce suppression of antitumor activity through the activation of ADO receptors expressed on immune cells [133]. Four subtypes of G protein-coupled-ADO receptors have been classified: A1R, A2AR, A2BR, and A3R [133]. T cells express on their surface mainly A2AR and A2BR [139,140]. By interacting with their receptors, ADO is able to potently inhibit proliferation, differentiation, cytokine secretion, and T-cell cytotoxic activity [140]. This mechanism is mediated by the accumulation of intracellular cAMP, which stimulates the activity of protein kinase A and of exchange protein directly activated by cAMP (EPAC) [140,141,142]. ADO stimulates regulatory T cells (Tregs) differentiation [140].

ADO also interferes with the differentiation of monocytes in DCs and with their activation. By the activity of A2AR and A2BR, it impairs the capability of DC to induce the Th1 immune response, whereas Th2 and Th17 responses are stimulated. ADO enhances the production of interleukins (ILs) IL-5, IL-6, IL-10, transforming growth factor- β (TGF-β), indoleamine-pyrrole 2,3-dioxygenase-1 (IDO-1), arginase 2, programmed cell death ligand 2 (PD-L2), and VEGF whereas it reduces the expression of tumor necrosis factor-α (TNF-α) and IL-12 [140,143,144,145].

The interaction between ADO and its receptors compromises the differentiation of monocytes into macrophages [140,146]. By stimulating the expression of arginase-1, IL-10, and VEGF, ADO prompts the macrophages toward the M2 phenotype [140,146].

The activation of A2AR by ADO impairs NK cells’ maturation, proliferation, and the secretion of interferon-γ (IFN-γ) and TNF-α. The stimulation of A2AR decreases the NK cell cytotoxic capability [140].

A2BR stimulation boosts IMC differentiation toward MDSCs [147]. A2AR-mediated signaling stimulates IL-10 secretion by MDSC cells [140]. An enhancement in CD73 expression was reported upon administration of an ADO analog [147].

## 5. microRNA in HCC Metabolism

microRNA (miRNAs) are small non-coding RNAs that play key roles in many cellular functions, including metabolism [148]. Several miRNAs have been found to be dysregulated in numerous malignancies contributing to tumorigenesis [49].

The liver-specific miR-122 exerts a key role in the sustenance of the physiological liver metabolism [149]. Alterations in its expression are often associated with liver dysfunctions and the development of liver malignancies [149]. miR-122 is downregulated in HBV-related HCC; this reduction in miR-122 expression is associated with a decreased mitochondrial metabolism in HCC and in non-pathological liver tissues [150]. miR-122 can be associated with morbidity and mortality of HCC patients [150]. miR-122, together with miR-26a and miR-130a, have been found to be negatively regulated in HCC, and the consequent upregulation of their target genes caused aberrant cell proliferation [151]. In addition, miR-122 targets PKM2 [149].

The miR-520 family is significantly downregulated by Tat-activating regulatory DNA-binding protein (TARDBP). High TARDBP levels have been found in HCC, where it regulates the expression of PFKP [152].

miR-7 acts as a tumor suppressor in HCC. Specifically, miR-7 reduces tumor cell proliferation and migration through PI3K/AKT/mTOR signaling [153].

Another regulator of the PI3K/AKT/mTOR pathway is miR-125a, which impairs cell proliferation and migration [154].

MCT4, responsible for lactate efflux, is targeted by miR-145 [155]. The regulation of MCT4 by miR-145 induces a significant downfall of the intracellular pH with damage of the pH homeostasis of the TME. Furthermore, the quick pH reduction of the intracellular compartment interferes with a wide spectrum of enzymes involved in glycolysis, subsequently hindering HCC proliferation and metastasis [155].

## 6. Targeted Therapies for Metabolic Pathways

Molecular pumps that move the pharmacological compounds across the plasma membranes of tumor cells are the major mechanisms that induce drug resistance [156]. Most of the molecular pumps belong to the ATP binding cassette (ABC) transporter family [156]. In HCC, permeability-glycoprotein MDR1, encoded by *ABCB1* gene, and breast cancer resistance protein (BCRP), encoded by *ABCG2* gene, need to be briefly mentioned. MDR1 is an ATP-binding cassette able to recognize a broad number of hydrophobic molecules, such as chemotherapeutic compounds, and extrudes them by ATP hydrolysis across the extracellular compartment [156]. Upregulation of MDR1 in sorafenib-resistant HCC cells was correlated with the EMT phenotype, PI3K signaling, and sorafenib-induced hypoxia [157,158]. MDR1 expression was significantly higher in invasive HCC compared to non-invasive HCC [159]. The BCRP mechanism of action for drug efflux is very similar to MDR1 [160]. *BCRP* mRNA expression was found to be upregulated in liver diseases and in HCC compared to normal tissues, particularly in less differentiated HCC [161]. The expression of BCRP is stimulated by HIF-2α, thus inducing anticancer drug resistance [162].

Disruption of KEAP1 renders HCC cells resistant to regorafenib and lenvatinib, with an increase in Nrf2 activity [163]. Nrf2 upregulation correlates with Bcl-xL levels [106].

Another drawback of current HCC therapies is the formation of a hypoxic environment [158,164,165,166,167,168,169]. Sorafenib-resistant HepG2 cells presented higher levels of HIF-1α and HIF-2α with nuclear localization with respect to sorafenib-responsive HepG2 cells [166]. Sorafenib can induce the activation of the HIF-2α pathway with the consequent stimulation of EGFR signaling [164]. HCC tissues of patients resistant to sorafenib showed an increase of HIF-1α levels with respect to HCC tissue samples of untreated patients. HCC patients still sensitive to sorafenib had a lesser increase of HIF-1α levels [158]. Increased expression levels of HIF-1α, MMP9, and mesenchymal markers were also detected in HCC tissues of patients previously treated with TACE with respect to the untreated ones [167]. 

During hypoxia, HIF-1α can also regulate the expression of the *MDR1* gene and MDR1 protein [170]. A positive correlation was found between HIF-1α and MDR1 levels in HCC cells subjected to prolonged administration of 5-fluorouracil. In addition, a negative correlation was detected between apoptosis index and HIF-1α/MDR1 expression [171]. HIF-1α can act on apoptosis by regulating two anti-apoptotic proteins: baculoviral IAP repeat containing 3 (BIRC3) and myeloid cell leukemia-1 (MCL-1) [172,173]. In glioblastoma multiforme, HIF-1α signaling stimulates the expression of BIRC3, which could have an active role in resistance to treatments arresting apoptosis and inducing a pro-inflammatory phenotype [172]. A similar behavior was detected in HCC cells, where an analysis revealed an hypoxia-responsive element for HIF-1 localized in the promoter region of MCL-1, which allows HIF-1 to exert its anti-apoptotic role [173,174].

**Figure 3:**
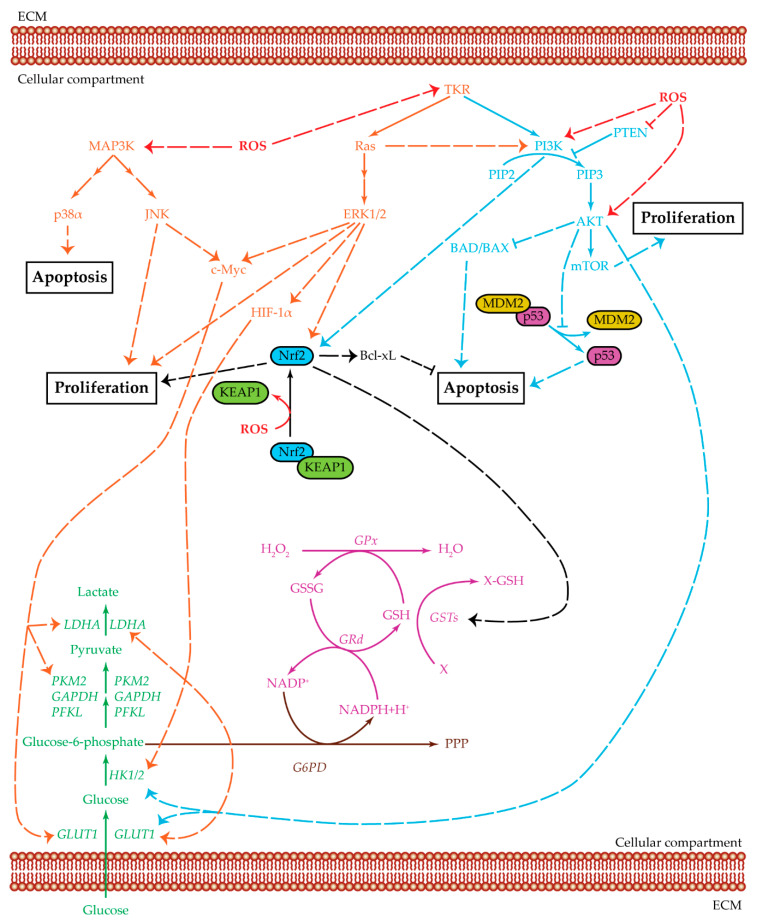
Crosstalk among glycolysis, MAPK, and PI3K pathways. Summarized are the three main pathways of cell proliferation and survival and the relevant cross-regulatory network: MAPK (orange), PI3K/AKT/mTOR (light blue), and glycolysis (green). Three related additional pathways are highlighted: glutathione cycle (purple), Nrf2 (black), and PPP (brown). Positive regulations are pictured as dotted arrows, and negative regulations are pictured as dotted truncated arrows. HCC cell survival is a complex mechanism that involves several cellular pathways which act both in single ways and as intersected signaling cascades. MAPK and PI3K/AKT/mTOR can be activated by tyrosine-kinase receptors to upregulate cell proliferation. They also act as inhibitors for cellular apoptosis. Ras protein, an effector of the MAPK pathway, can positively regulate PI3K, which increases AKT levels. AKT, in turn, upregulates both HK2 and GLUT1, thus favoring the glycolysis cascade. PI3K and ERK1/2 can positively regulate Nrf2, thus further increasing the signals for cellular proliferation. The upregulation of Nrf2 increases the GST levels which, in turn, maintains the ROS balance in a state favoring cell survival. The GST enzyme is part of the glutathione cycle and it acts with GSH as detoxification agent against oxidative byproducts. GSH and GSSG levels are regulated by GPx, an enzyme mainly used to convert H_2_O_2_ into the harmless H_2_O, and GRd, which uses NADPH to reduce GSSG and refill the GSH pool. The MAPK pathway, acting through JNK and ERK1/2, positively regulates c-Myc, which in turn upregulatesGLUT1, PKM2, and LDH expression levels. ERK1/2 can also act through HIF-1α to upregulate GLUT1, HK2, and LDH. Therefore, both JNK and ERK1/2 have a positive effect on the glycolytic pathway, favoring glucose entrance and lactate production. Apoptosis is inhibited in several ways: Nrf2 is positively associated with Bcl-xL, AKT inhibits both BAD/BAX and the dissociation between MDM2 and p53, p38α is present at low levels in HCC samples. In this proliferative environment, ROS can act in several ways: favoring MAPK cascade, positively regulating PI3K and AKT, inhibiting PTEN action, and enabling the dissociation of KEAP1 from Nrf2, which is then able to bind DNA and act as transcription factor. Abbreviations: AKT = protein kinase-B; BAD/BAX = Bcl-2 associated agonist of cell death/bcl-2-like protein 4; Bcl-xL = B-cell lymphoma-extra-large protein; c-Myc = Myc proto-oncogene protein; ECM = extracellular matrix; ERK1/2 = extracellular regulated kinases 1 and 2; G6PD = glucose-6-phosphate dehydrogenase; GAPDH = glyceraldehyde-3-phosphate dehydrogenase; GLUT1 = glucose transporter 1; GPx = glutathione peroxidase; GRd = glutathione reductase; GSH = glutathione reduced form; GSSG = glutathione oxidized form; GSTs = glutathione S-transferases; HIF-1α = hypoxia inducible factor α; HK1/2 = hexokinase 1/2; JNK = c-Jun N-terminal kinases; KEAP1 = Kelch-like ECH-associated protein 1; LDHA = lactate dehydrogenase A; MAP3K = MAPK kinase kinase; MDM2 = mouse double minute 2 homolog; mTOR = mammalian target of rapamycin; Nrf2 = nuclear factor erythroid 2-related factor 2; p38α = p38 mitogen-activated protein kinase α; p53 = cellular tumor antigen p53; PFKL = phosphofructokinase L; PI3K = phosphatidylinositol 3-kinase; PIP2 = phosphatidylinositol 4,6-bisphosphate; PIP3 = phosphatidylinositol 3,4,5-triphosphate; PKM2 = pyruvate kinase isoenzyme M2; PPP = pentose phosphate pathway; PTEN = phosphatase and tensin homologue; Ras = rat sarcoma protein; ROS = reactive oxygen species; TKR = tyrosine-kinase receptor; X = oxidative stress byproduct; X-GSH = oxidative stress by-product bound to GSH.

Three main pathways are upregulated in HCC due to a metabolic switch and elevated ROS production: glycolysis, MAPK pathway, and PI3K/AKT/mTOR pathway, which can interact with one another to increase their effects in tumor proliferation and survival (Figure 3) [82,94,175]. Combinatorial treatments targeting both MAPK pathway and PI3K/AKT/mTOR pathway have been proposed to block tumor proliferation and promoting cellular apoptosis [175,176,177,178].

On clinicaltrials.gov, there are some completed trials regarding the efficacy of treatment combinations using PI3K and MAPK inhibitors as anti-cancer therapies, although without straightforward results [179,180,181,182,183].

Long-term sorafenib exposure causes chemoresistance with an upregulation in the PI3K/AKT/mTOR pathway [184,185]. Based on this evidence, different PI3K pathway inhibitors (PI3Kpi) have been proposed to be used in combination with sorafenib [157,178,184,185,186,187,188,189]. 

PI3K and MAPK pathways are regulators of anaerobic glycolysis [190]. The first one upregulates GLUT1 and HK through AKT activity, while the second activates several transcription factors through ERK1/2, in particular c-Myc which can increase GLUT1, LDHA, and PKM2 expression, and HIF-1α, which upregulates GLUT1, HK2, and LDHA [13,65,67,82,190]. Treatment combinations capable of downregulating PI3K and MAPK pathways can also inhibit cellular proliferation and survival by reducing the glycolytic rate [190]. Another possibility is to inhibit one of the two pathways and glycolysis itself, in particular, by glucose deprivation and treatment with PI3Kpi [191,192,193]. The treatment combination of 2-deoxyglucose (2-DG), G6P competitor, and sorafenib increased HCC cell death, inhibited colony formation, decreased cell motility and MMP9 expression, and had tumor-suppression effects [194,195].

An alternative strategy to treat HCC is to increase ROS levels in order to augment oxidative stress [11]. The sorafenib–diclofenac combination showed interesting results regarding HCC cell death [196]. In vitro studies on HCC cell lines treated with sorafenib and ethacrynic acid, a GST inhibitor, suggested GST as a possible co-target [197].

Another possible approach is to act on the hypoxic TME. LS081 was shown to inhibit cell growth through prolyl hydroxylation of HIF-1α and its degradation [198]. Gefitinib inhibited the HIF-2α-induced proliferation of HCC cells acting on EGFR. Gefitinib had a synergistic effect with sorafenib to induce apoptosis [164]. Treatment with a siRNA targeting HIF-2α showed a downregulation of VEGF, HIF-2α, and TGF-α [164]. EF24, a molecule with a structure like curcumin, was able to upregulate VHL which, in turn, mediated HIF-1α degradation. HCC cells treated with EF24 showed an inhibition of cell viability and metastasis, and an increase in sorafenib-induced apoptosis [158]. Short hairpin RNA-HIF-2α in combination with sorafenib was shown to inhibit HCC cell proliferation in a hypoxic environment [168]. Evofosfamide is a hypoxia-activated prodrug. In normoxic conditions, evofosfamide is not toxic, while, in a hypoxic environment, it is activated and introduces intra- and inter-strand crosslinks in DNA [169,199]. In rabbits bearing the HCC VX2 tumor model, the treatment combination of TACE and evofosfamide showed enhanced antitumor efficacy with respect to TACE or evofosfamide alone [169]. Evofosfamide was tested in a phase Ib trial in combination with sorafenib in patients with advanced HCC [200]. A list of the proposed combination treatments tested in vitro and/or in vivo is reported in Table 1.

To date, on ClinicalTrials.gov, there are several ongoing clinical trials based on small-molecule tyrosine-kinase inhibitors capable of altering HCC metabolism, and thus inhibiting tumor growth or recurrence. These trials are listed in Table 2.

## 7. Conclusions and Future Perspectives

Despite recent advances in therapeutic options, curative therapies for HCC are still not available [1,2,201,202,203,204,205,206,207,208,209,210]. HCC is a tumor type with a well-known susceptibility to mutate under selective pressure, such as that exerted by anti-cancer drugs. This implies an associated drug resistance based on a plethora of mechanisms [211,212,213,214,215,216,217,218]. One of the peculiar features of cancer is the metabolic reprogramming [7,219,220,221]. Thus, targeting of tumor metabolism pathways could represent a useful therapeutic strategy for HCC treatment [156,157,158,163,222]. This treatment approach could be of particular interest if included in the context of chemo- and/or immuno-treatment combinations [223,224]. An example of a treatment combination targeting both immune and metabolic pathways is the IMbrave150 clinical trial, which showed promising results for unresectable HCC previously untreated with systemic therapy. The study compared the outcomes of sorafenib treatment versus atezolizumab (anti-PDL1) plus bevacizumab (anti-VEGF). Combination therapy showed better OS rates at 6 and 12 months: 84.8% and 67.2%, respectively, in comparison to sorafenib group (72.2% and 54.6%, respectively). Patients treated with combination therapy showed a longer progression-free survival than the sorafenib arm: 6.8 months versus 4.3 months, respectively. Patients who received combination therapy had a delayed decline in their quality of life with respect to sorafenib-treated patients: 11.2 months versus 3.6 months, respectively [225].

To extend the impact of immunotherapy to more patients, targeting additional mechanisms of tumor immune evasion will be critical [226]. The biogenesis of lactic acid could represent an interesting target for the development of novel therapeutic approaches [227]. The development of combinatorial strategies that include agents targeting the immunosuppression mediated by adenosine could significantly increase the efficacy of immunotherapeutic approaches [140].

## Figures and Tables

**Table 1:** Treatment combinations tested in vitro in HCC cell lines and in vivo in HCC animal models.

Drug Combination.	Pathways Involved	In Vitro Results	In Vivo Results	Reference
Sorafenib + MK-2206	MAPK + PI3K	Reversion of EMT and MDR1.		[157]
Sorafenib + PI-103	MAPK + PI3K	Inhibition of proliferation; blockage of MAPK and PI3K pathways.	Inhibition of tumor growth; increase in apoptosis.	[178,187]
Sorafenib + LY294002	MAPK + PI3K	Decrease in cell viability; increase in pro-apoptotic proteins; EMT inhibition.		[184]
Sorafenib + copansilib	MAPK + PI3K	Antineoplastic effect.		[185]
Sorafenib + BEZ235	MAPK + PI3K	Inhibition of proliferation and migration; increase in apoptosis.		[186]
Sorafenib + CMG002	MAPK + PI3K	Downregulation of pAKT and pERK.	Inhibition of tumor growth; increase in apoptosis.	[188]
Sorafenib + rapamycin	MAPK + PI3K	Decrease in proliferation; increase in apoptosis.	Tumor necrosis and skin ulceration.	[189]
Sorafenib + 2-DG	MAPK + Glycolysis	Massive cell death; inhibition of colony formation.	Tumor suppression.	[194]
Sorafenib + 2-DG	MAPK + Glycolysis	Decrease in proliferation; decrease in motility; decrease in *MMP9* expression.		[195]
Sorafenib + diclofenac	MAPK + ROS	Increase in cell death; decrease in GSH and increase in ROS.	Decrease in tumor burden.	[196]
Sorafenib + ethacrynic acid	MAPK + ROS	Effect on cell viability.		[197]
Sorafenib + EF24	MAPK + HIF-1α	Growth inhibition; inhibition of migration and invasion.	Decrease in metastasis formation; inhibition of tumor growth; increase in apoptosis.	[158]
Sorafenib + gefitinib	MAPK + HIF-2α	Inhibition of proliferation and induction of apoptosis.		[164]
Sorafenib + HIF-2α siRNA	MAPK + HIF-2α	Decrease of cell viability and increase in apoptosis.	Inhibition of tumor growth; increase in apoptosis.	[164]
Sorafenib + short hairpin RNA-HIF-2α	MAPK + HIF-2α	Antiproliferative activity.	Inhibition of tumor growth.	[168]

**Table 2:** Ongoing clinical trials investigating the use of tyrosine-kinase inhibitors for hepatocellular carcinoma treatment.

Molecule Name	Study Title	Status	Drugs	Phase	NCT Number
Sorafenib	YIV-906 (Formerly PHY906/KD018) With Sorafenib in HBV(+) Hepatocellular Carcinoma (HCC)	Recruiting	Drug: YIV-906 plus Sorafenib Drug: Placebo plus Sorafenib	II	NCT04000737
	Sorafenib Plus TACE Versus Sorafenib Alone as Postoperative Adjuvant Treatment for Resectable Primary Advanced HCC	Recruiting	Drug: Sorafenib Procedure: Transarterial chemoembolization	III	NCT04143191
	HAIC Plus Toripalimab vs. HAIC Plus Sorafenib for HCC With PVTT: a Non-comparative, Prospective, Randomized Trial	Recruiting	Procedure: Hepatic arterial infusion chemotherapy Drug: Toripalimab Drug: Sorafenib	II	NCT04135690
	SBRT+TACE+Sorafenib Vs Sorafenib in the Treatment of uHCC With PVTT	Recruiting	Radiation: SBRT+TACE+Sorafenib Drug: Sorafenib	III	NCT04387695
	Sorafenib Combined With Arsenical in Treating Patients With Recurrent HCC After Liver Transplantation	Recruiting	Drug: Arsenical Drug: Sorafenib	II	NCT04232722
Regorafenib	Regorafenib Followed by Nivolumab in Patients With Hepatocellular Carcinoma (GOING)	Recruiting	Drug: Regorafenib Drug: Nivolumab	I and II	NCT04170556
Lenvantinib	Efficacy and Safety of Lenvatinib as an Adjuvant Therapy for Hepatocellular Carcinoma	Recruiting	Drug: Lenvima 4 mg Oral Capsule	II	NCT04227808
	Immunotherapy With Nivolumab in Combination With Lenvatinib for Advanced Stage Hepatocellular Carcinoma	Recruiting	Drug: Lenvatinib Drug: Nivolumab	II	NCT03841201
	Preliminary Antitumor Activity, Safety and Tolerability of Tislelizumab in Combination With Lenvatinib for Hepatocellular Carcinoma	Recruiting	Drug: Lenvatinib Drug: Tislelizumab	II	NCT04401800
	A Study of CS1003 in Subjects With Advanced Hepatocellular Carcinoma	Recruiting	Drug: CS1003 plus Lenvatinib Drug: CS1003 Placebo plus Lenvatinib	III	NCT04194775
	HAIC Plus Lenvatinib and Toripalimab for Advanced HCC	Recruiting	Procedure: Hepatic arterial infusion chemotherapy Drug: Lenvatinib Drug: Toripalimab	II	NCT04044313
	Systemic Chemotherapy Plus Lenvatinib and Toripalimab for HCC With Extrahepatic Metastasis	Recruiting	Procedure: Systemic chemotherapy Drug: Lenvatinib Drug: Toripalimab	II	NCT04170179
	PD-1 Monoclonal Antibody, Lenvatinib and TACE in the Treatment of HCC	Recruiting	Combination Product: PD-1 mAb combined with TACE and Lenvatinib	II	NCT04273100
	TACE With Lenvatinib Versus Lenvatinib Alone in in First-line Treatment of Advanced HCC	Recruiting	Procedure: TACE Drug: Lenvatinib	III	NCT03905967
	Efficacy and Safety of Lenvatinib as a Conversion Therapy for HCC	Recruiting	Drug: Lenvatinib	II	NCT04241523
	Safety and Efficacy of Lenvatinib (E7080/MK-7902) With Pembrolizumab (MK-3475) in Combination With Transarterial Chemoembolization (TACE) in Participants With Incurable/Non-metastatic Hepatocellular Carcinoma (MK-7902-012/E7080-G000-318/LEAP-012)	Recruiting	Drug: Lenvatinib Biological: Pembrolizumab Drug: Oral Placebo Drug: IV Placebo Procedure: TACE	III	NCT04246177
	A Study of E7386 in Combination With Other Anticancer Drug in Participants With Solid Tumor	Recruiting	Drug: E7386 Drug: Lenvatinib	I	NCT04008797
Cabozantinib	Cabozantinib toLERANCE Study in HepatoCellular Carcinoma (CLERANCE)	Recruiting	Drug: Cabozantinib group Other: ECG	IV	NCT03963206
Brivanib	MGD013 Monotherapy and Combination With Brivanib Dose Escalation and Expansion Study in Advanced Liver Cancer Patients	Recruiting	Drug: MGD013 monotherapy Drug: MGD013 in combination with Brivanib Alaninate	I and II	NCT04212221
Apatinib	SHR-1210 Combined With Apatinib Mesylate in the Perioperative Treatment of Hepatocellular Carcinoma	Recruiting	Drug: Apatinib Combined With SHR-1210 Injection	II	NCT04297202
	SHR-1210 Plus Apatinib in Patients With Advanced-Stage Hepatocellular Carcinoma	Recruiting	Drug: SHR-1210 Drug: Apatinib	II	NCT04014101
	A Trial of Hepatic Arterial Infusion Combined With Apatinib and Camrelizumab for C-staged Hepatocellular Carcinoma in BCLC Classification	Recruiting	Combination Product: Hepatic Arterial Infusion combined with Apatinib and Camrelizumab	II	NCT04191889
	Combination Camrelizumab (SHR-1210) and Apatinib for Downstaging/Bridging of HCC Before Liver Transplant	Recruiting	Drug: Camrelizumab plus Apatinib	I and II	NCT04035876
	The Safety and Efficacy of Thermal Ablation Combined With Apatinib and Carilimub for Advanced Liver Cancer	Recruiting	Drug: Apatinib Mesylate Drug: SHR-1210	II	NCT04204577
	RFA Plus Carrizumab and Apatinib vs Carrizumab and Apatinib Alone for HCC	Recruiting	Combination Product: radiofrequency ablation plus Carrizumab and Apatinib Combination Product: Carrizumab and Apatinib	II	NCT04150744
	A Study to Evaluate SHR-1210 in Combination With Apatinib as First-Line Therapy in Patients With Advanced HCC	Recruiting	Drug: SHR-1210 Drug: Apatinib Drug: Sorafenib	III	NCT03764293

Summarized are the ongoing clinical trials found on ClinicalTrials.gov by searching the keywords “hepatocellular carcinoma” and “each small molecule name”. The research has been done adding the following filters: “Active, not recruiting”; “Recruiting”; “Study starts from 06/01/2019”. Trials with the principal small molecule used only as a comparative drug are not included.
